# Insulin, Central Dopamine D2 Receptors, and Monetary Reward Discounting in Obesity

**DOI:** 10.1371/journal.pone.0133621

**Published:** 2015-07-20

**Authors:** Sarah A. Eisenstein, Danuta M. Gredysa, Jo Ann Antenor–Dorsey, Leonard Green, Ana Maria Arbeláez, Jonathan M. Koller, Kevin J. Black, Joel S. Perlmutter, Stephen M. Moerlein, Tamara Hershey

**Affiliations:** 1 Psychiatry Department, Washington University in St. Louis, St. Louis, MO, United States of America; 2 Radiology Department, Washington University in St. Louis, St. Louis, MO, United States of America; 3 Psychology Department, Washington University in St. Louis, St. Louis, MO, United States of America; 4 Pediatrics Department, Washington University in St. Louis, St. Louis, MO, United States of America; 5 Neurology Department, Washington University in St. Louis, St. Louis, MO, United States of America; 6 Anatomy and Neurobiology Department, Washington University in St. Louis, St. Louis, MO, United States of America; 7 Programs in Physical Therapy and Occupational Therapy, Washington University in St. Louis, St. Louis, MO, United States of America; 8 Biochemistry and Molecular Biophysics Department, Washington University in St. Louis, St. Louis, MO, United States of America; Duke University Medical Center, UNITED STATES

## Abstract

Animal research finds that insulin regulates dopamine signaling and reward behavior, but similar research in humans is lacking. We investigated whether individual differences in body mass index, percent body fat, pancreatic β-cell function, and dopamine D2 receptor binding were related to reward discounting in obese and non-obese adult men and women. Obese (n = 27; body mass index>30) and non-obese (n = 20; body mass index<30) adults were assessed for percent body fat with dual-energy X-ray absorptiometry and for β-cell function using disposition index. Choice of larger, but delayed or less certain, monetary rewards relative to immediate, certain smaller monetary rewards was measured using delayed and probabilistic reward discounting tasks. Positron emission tomography using a non-displaceable D2-specific radioligand, [^11^C](*N*-methyl)benperidol quantified striatal D2 receptor binding. Groups differed in body mass index, percent body fat, and disposition index, but not in striatal D2 receptor specific binding or reward discounting. Higher percent body fat in non-obese women related to preference for a smaller, certain reward over a larger, less likely one (greater probabilistic discounting). Lower β-cell function in the total sample and lower insulin sensitivity in obese related to stronger preference for an immediate and smaller monetary reward over delayed receipt of a larger one (greater delay discounting). In obese adults, higher striatal D2 receptor binding related to greater delay discounting. Interestingly, striatal D2 receptor binding was not significantly related to body mass index, percent body fat, or β-cell function in either group. Our findings indicate that individual differences in percent body fat, β-cell function, and striatal D2 receptor binding may each contribute to altered reward discounting behavior in non-obese and obese individuals. These results raise interesting questions about whether and how striatal D2 receptor binding and metabolic factors, including β-cell function, interact to affect reward discounting in humans.

## Introduction

Human obesity is associated with altered dopamine (DA) function [1], altered DA D2-like receptor binding in brain reward regions [2–3], altered reward-related behavior [4–6], and insulin dysregulation [7–8]. How these factors relate to each other remains unclear. Understanding the precise relations among dopaminergic dysfunction, altered reward behavior, and metabolic factors may be critical for identifying behavioral subtypes of obesity, and for specifying targets of clinical intervention along the complex pathway linking neuroendocrine hormones and behavior.

In nonhuman animals, the pancreatic β-cell-secreted hormone insulin binds directly to insulin receptors located on DA neurons in brain reward pathways [9], and regulates DA signaling, reward processing, and reward behavior by increasing DA transporter (DAT) density and function in the striatum [10–12]. Insulin also interacts with D2-like receptors to affect DA-dependent behavior [13], increases brain reward thresholds [14], and reduces preference and operant responding for food reward [15–16], non-hedonic food intake [17], and hedonic food intake in sated animals [11].

Recent human neuroimaging evidence indicates that insulin alters brain activity and response to food cues in healthy individuals [18–19]. Specifically, insulin administration decreases palatable food intake [20–21], and oral glucose-induced insulin reactivity lessens brain activation in response to images of food [22]. In insulin-resistant individuals, the increased rate of brain glucose metabolism normally associated with insulin infusion is decreased, particularly in regions related to reward such as ventral striatum [23]. Lower insulin sensitivity is associated with increased brain activation induced by food images [24] and increased striatal D2-like receptor binding [3]. Finally, in obese but not lean individuals, activity in reward-related brain regions mediates the relation between insulin resistance and food craving [25]. To our knowledge, however, there are no published human studies of the relations among pancreatic β-cell insulin secretion, striatal D2 receptor (D2R) binding, and food or non-food reward discounting behavior in the same individual.

The goal of the present study was to determine specific relations among body mass index (BMI), percent body fat (PBF), β-cell function, D2R binding, and monetary reward discounting behavior in obese and non-obese adults. Steep discounting of delayed rewards is associated with increased impulsivity and poor self-control whereas shallow discounting of probabilistic rewards is related to increased risk-taking behavior [26], characteristics that may fuel problem eating behavior in obesity. We hypothesized that 1) higher BMI and PBF, 2) lower β-cell function, as reflected by disposition index (DI) values, and 3) lower striatal D2R binding, would relate to greater preference for an immediate, smaller (greater discounting of a delayed reward, DRD) and larger but less certain (less discounting of a less probable reward, PRD) monetary reward in both non-obese and obese groups. The direction of the latter hypothesis is based on animal and human studies that indicate associations between decreased striatal D2-like receptor availability and addiction-like behavior and/or obesity [1,27–29]. Striatal D2R specific binding was quantified with [^11^C] (*N*-methyl)benperidol ([^11^C]NMB), a novel radioligand that, unlike other commonly used D2-like receptor PET radioligands, is over 200-fold selective for D2R over D3 receptors [30], is not displaced by endogenous DA [31], making it suitable for measuring absolute D2R binding. We further predicted that lower β-cell function, but not BMI [32] or PBF, would relate to decreased D2R binding in non-obese and obese groups.

## Materials and Methods

### Participants

Volunteers were assessed with a detailed history, including neurological and physical examinations, psychiatric interviews [33], and routine blood tests (e.g., fasting plasma glucose, lipids, serum creatinine, hematocrit). Individuals were excluded for history of medical problems (e.g., diabetes) as well as other significant neurological, cerebrovascular, cardiovascular, or psychiatric diagnosis (DSM-IV Axis I disorders except for specific phobias), head trauma, any current or recent dopaminergic drug use (e.g., stimulants, agonists, bupropion, neuroleptics or metoclopramide), current heavy alcohol use (males >2 drinks per day, females >1 drink per day) or illicit drug use [34], history of substance abuse or dependence, or IQ below 70 as measured by the Wechsler Adult Intelligence Scale [35]. All women were premenopausal. Fifteen non-obese and 15 obese participants overlap with the sample reported on previously [32].

### Ethics Statement

The study (IRB ID#201104109) was approved by the Washington University School of Medicine Human Research Protection Office and the Radioactive Drug Research Committee, and was carried out in accordance with the principles expressed in the Declaration of Helsinki. All participants gave written informed consent prior to participation.

### Obesity and insulin measures

An average of 13.9 days (S.D. = 17.1) prior to the positron emission tomography (PET) scans and usually on the same day that reward discounting was assessed, BMI and PBF were obtained by dual-energy X-ray absorptiometry using the GE Lunar iDXA (GE Healthcare; Chalfont St Giles, UK; [36]) (1 person completed the reward discounting task 1 month after the PET scan and another completed the task on the day of the PET scan). Participants also underwent a 2-hour oral glucose tolerance test (OGTT), with arterialized hand vein sampling of insulin, C-peptides, and blood glucose levels at times –5, 0, 10, 20, 30, 60, and 120 minutes after drinking a standard 75g glucose load. The oral glucose minimal model provides a measure of insulin sensitivity that compares well with insulin sensitivity estimated from an intravenous glucose tolerance test [37]. Pancreatic β-cell function was estimated using this model to calculate a DI (Disposition Index = insulin sensitivity × insulin secretion for the given amount of glucose). Unlike the homeostatic model assessment of insulin resistance (HOMA-IR), which is based solely on fasting levels, this index is a more comprehensive measure of whole body insulin sensitivity that takes into account both the fasting and post-glucose load values [38]. Whole-body insulin sensitivity was estimated using the Matsuda insulin sensitivity index (Matsuda ISI; 10,000/√[(Glucose_t0’_ (mg/dL) × Insulin_t0’_ (mU/L) × (Glucose_mean_ × Insulin_mean_)] [39]; a higher Matsuda ISI indicates greater insulin sensitivity. Postprandial β-cell insulin secretion was calculated using the minimal model analysis, providing an index (Phi Total) of insulin secretion in relation to a plasma glucose concentration that relies on plasma C-peptide as a function of glucose concentration [38]; higher Phi Total indicates greater pancreatic β-cell secretion of insulin in response to glucose load. DI was selected as the primary insulin measure of interest because it accounts both for how much insulin is secreted for a given amount of ingested glucose, and for how effective insulin secretion is at clearing glucose [38]. A higher DI indicates better β-cell function.

### PET & MRI acquisition, preprocessing, and analyses

Structural magnetic resonance T1-weighted anatomical images were acquired on a Siemens Magnetom Tim Trio 3T scanner using a 3-D MP-RAGE sequence (sagittal orientation, TR = 2400 ms, TE = 3.16 ms, flip angle = 8 degrees, slab thickness 176mm, FOV = 256x256mm; voxel dimensions = 1x1x1 mm). PET images were acquired on a Siemens/CTI ECAT EXACT HR+ scanner using ([^11^C]NMB). [^11^C]NMB was prepared using an automated system based on published methods [40–41]. Benperidol (Janssen Pharmaceutica) was [^11^C]methylated with [^11^C]CH_3_I made with the Washington University JSW BC-16/8 cyclotron and GE PETtrace MeI MicroLab, and product [^11^C]NMB was isolated using preparative HPLC. The radiopharmaceutical was terminally sterilized by membrane filtration (0.2 μM) and reformulated in 10% ethanol in Sodium Chloride for Injection, USP. The product radiochemical purity exceeded 95%, and specific activity ≥ 2000 Ci/mmol (74 TBq/mmol). In all studies, the injected dose of unlabeled NMB was ≤ 7.3 μg. Each participant received 6.4–18.1 mCi [^11^C]NMB intravenously.

ROIs including the dorsal striatum (putamen, caudate), and ventral striatum (nucleus accumbens (NAc)) were selected *a priori* and identified using FreeSurfer [42]. To reduce partial volume effects, putamen and caudate regions were eroded by approximately 2 mm from the surface by combining a Gaussian smoothing filter with thresholding. The NAc volume was not large enough to erode in this manner. For each participant, the dynamic PET images were co-registered to each other and to the participant’s MP-RAGE image, as described previously [43]. Striatal ROIs and the cerebellar cortex reference region were resampled in the same atlas space [44], and decay-corrected tissue activity curves were extracted for each ROI from the dynamic PET data. Non-displaceable binding potentials (BP_ND_) for DA D2R were determined for each ROI using the Logan graphical method with the whole cerebellum as the reference region [45]. D2R BP_ND_s for putamen, caudate, and NAc were averaged across left and right hemispheres to reduce the number of comparisons. The independent variable ‘Striatal D2R BP_ND_’ was calculated by summing BP_ND_s for putamen, caudate, and NAc in each individual.

### Behavioral paradigms and analyses

Participants completed delayed (DRD) and probabilistic (PRD) reward discounting tasks. Performance on these tasks may relate to dopaminergic signaling [46–47]. In addition, these tasks are associated with distinct neural mechanisms [48–49] and decision-making processes in humans [50–51], and were used in previous studies of human obesity [6,52–53]. Participants first completed 2 separate practice rounds (1 for DRD and 1 for PRD), consisting of 5 trials each, before completing the actual tasks. Presentation order regarding type of task was randomly assigned. The DRD task consisted of 5 trials per delay condition in which participants made a series of choices, indicated by mouse click, between two hypothetical monetary rewards displayed simultaneously on the computer screen: an immediate, smaller amount (to be received “now”), and a larger, delayed amount to be received in the future at each of 5 delays (1 week, 1 month, 6 months, 1 year, and 2 years from now). The smaller immediate reward amount varied from trial to trial but the delayed larger reward was held constant at $500. The PRD task was identical to the DRD task except that the probability of receiving the larger amount varied (10%, 25%, 50%, 75%, or 90%) instead of time to obtaining the reward. For each participant, the presentation order of the 5 different delay and probability conditions was randomized within each task. Visual locations for choices were randomly positioned on the left and right side of the screen.

For each delay condition and for each probability condition, a series of “indifference points” was computed in an iterative fashion, representing the points at which the immediate/certain and delayed/probabilistic amounts were of approximately equal subjective value for an individual. For the first choice trial within a delay or a probability condition, the small, immediate/certain amount ($250) was always half of the delayed/less probable larger reward amount. For each subsequent trial within a condition, the immediate/certain, smaller amount was half the size of the previous change; the immediate/certain amount increased or decreased in the direction of the participant’s previous choice. Completion of the tasks yielded 5 indifference points each for DRD and PRD. Degree of reward discounting in an individual was determined by calculating area under the curve (AuC) [54], an atheoretical measure of the degree to which a reward decreases in subjective value as a function of delay (DRD_AuC_) or probability (“odds against;” PRD_AuC_) [55]. AuC values range from 0.0 (complete discounting) to 1.0 (no discounting). Lower AuC values indicate greater discounting as a function of delay or odds against; that is, lower DRD_AuC_ reflects greater preference for immediate, smaller rewards, and higher PRD_AuC_ reflects greater preference for risky, larger rewards.

### Primary statistical analyses

Planned data analyses were conducted using SPSS v. 20.0. For each variable, distribution normality was assessed with one-sample Kolmogorov-Smirnov tests. Comparisons between non-obese and obese individuals were conducted using independent samples *t*-tests, Mann-Whitney *U* (for non-normally distributed variables), or, in the cases of DI, Matsuda ISI, Phi Total, striatal D2R BP_ND_, and reward discounting, analyses of covariance (ANCOVA) covarying for age, education level, sex, and ethnicity. Since only three individuals had ethnicities other than White or Black, ethnicity was entered as a binary variable—‘White or not.’ Differences in gender and ethnic distributions between non-obese and obese participants were assessed with Chi-Square tests. Mixed repeated measures ANOVAs determined whether subjective values of delayed or probabilistic rewards decreased at the same rate in non-obese and obese groups. Bivariate correlations were calculated as Pearson’s *r*.

Within-group analyses used hierarchical multiple linear regression models with appropriate covariates in Step 1 (i.e., age, gender, education, ethnicity, group (group was covaried in total sample analyses only)). Age and education were covaried because they have been shown to correlate with our variables of interest in previous studies [32, 56–58]. Step 2 of the model included a single predictor of interest: BMI, PBF, DI, or striatal D2R binding. The dependent variable was DRD_AuC_ or PRD_AuC_ except for the hierarchical multiple linear regression analyses testing relations among predictor variables. Small group sizes precluded use of an interaction term (i.e., group x BMI) in these analyses; therefore, regression analyses were performed separately in the total sample, non-obese, and obese groups. These separate group analyses were treated as exploratory and results were corrected for multiple comparisons by the Bonferroni method (Bonferroni-corrected *α* = 0.025). Partial correlations (*pr*) were calculated for each hierarchical linear regression model to describe the unique variance explained by each predictor variable and outcome variable. These analyses were also performed including only female participants.

We also explored the effects of β-cell function on variables of interest by examining relations with Matsuda ISI and Phi Total separately, but only in cases where the relation with DI was significant, to minimize the problem of multiple comparisons. Likewise, significant relations with striatal D2R binding were followed up with examining those between specific ROIs (putamen, caudate, Nac) and the outcome measure.

A threshold of *p*≤0.05 was used for significance testing except for separate group hierarchical multiple linear regression analyses, where α≤0.025 due to Bonferroni correction. Cohen’s effect size calculations for differences between groups (Cohen’s *d*; [59]) and for each hierarchical linear regression (Cohen’s *f* ^2^; [59]) were completed using StatCalc3 [60]. Other effect size statistics (*r*, *ƞ* ^2^_*p*_, *φ*) were calculated by hand or in SPSS.

## Results

### Participants

Twenty-seven obese adults and 20 non-obese adults participated. Two participants with BMI > 25 kg/m^2^ (25.9 and 25.1 kg/m^2^) were included in the non-obese group. All other participants included in the non-obese group could therefore be considered true ‘normal-weight’. There were no PET data for three obese participants due to attrition between behavioral testing and the PET scan day. The final sample for analyses involving PET data included 24 obese (4 male) and 20 non-obese (5 male) adults; for analyses involving DRD and PRD, data included 26 obese (4 male) and 19 non-obese (5 male) adults.

### Group comparisons

**Fig 1:**
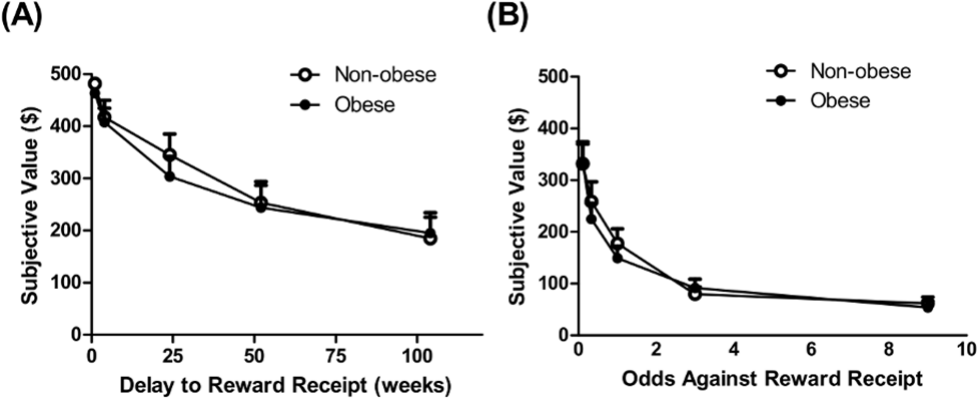
Non-obese and Obese Individuals Show Similar Reward Discounting Tendencies. (A) The subjective value of a monetary reward ($500) decreased as time to its receipt increased (main effect of time: *F*_4,120_ = 54.10, *p* < .001) in a similar manner in non-obese and obese individuals (no main effect of group: *F*_1,30_ = .12, *p* = 0.73 or group x time interaction (*F*_4,120_ = .36, *p* = 0.84). (B) The subjective value of a monetary reward ($500) decreased as the odds against its receipt increased (main effect of time: *F*_4,120_ = 88.66, *p*<0.001) in a similar fashion in non-obese and obese groups (no main effect of group: *F*_1,30_ = .12, *p* = 0.73 or group x time interaction (*F*_4,120_ = .67, *p* = 0.62).

Descriptive statistics for demographics and all study variables are summarized in **Table 1**. Non-obese and obese individuals differed significantly in BMI and PBF but not years of education or age. The non-obese and obese groups did not differ in gender distribution, but ethnicity distribution was different at a marginally significant level. β-cell function (DI) and insulin sensitivity (Matsuda ISI) were lower, and insulin secretion was higher, in obese relative to non-obese participants. Consistent with an overlapping sample in a previous publication from our lab [32], obese and non-obese groups did not differ significantly in striatal D2R BP_ND_. Reward discounting behavior did not differ between the non-obese and obese groups on either delay discounting (DRD_AuC_) or probabilistic discounting (PRD_AuC_ (**Fig 1**). DRD_AuC_ and PRD_AuC_ values were positively correlated across the total sample (*r*_45_ = .43, *p*<0.01), as well as within the non-obese (*r*_19_ = .51, *p* = 0.03) and obese (*r*_26_ = .39, *p* = 0.05) groups, such that greater preference for smaller and immediate monetary reward was associated with greater preference for smaller and certain ones.

**Table 1:** Participant characteristics.

	Obese (*n* = 27)		Non-obese (*n* = 20)		Group Comparisons		
	Mean (S.D.)	Range	Mean (S.D.)	Range	Test Statistic	*p*-value	Effect Size
**BMI** (kg/m^2^)	39.90 (4.76)	33.2–51	22.42 (2.40)	18.6–27.7	*U*_45_ = 217	<0.001***	0.85
**PBF**	48.67 (4.04)	39.7–55.6	32.74 (5.87)	20.8–43.6	*U*_45_ = 217	<0.01**	0.83
**Age** (years)	31.5 (6.61)	20–40.9	28.64 (5.28)	21.0–39.7	*U*_45_ = 408	0.12	0.23
**Education** (years)	14.96 (1.91)	12–18	15.90 (1.39)	13–18	*U*_45_ = 578	0.11	0.22
**Disposition Index**	123.68 (71.95)	54.4–288.8	276.61 (172.24)	74.6–758.4	*F*_1,41_ = 24.22	<0.001***	0.37
Matsuda ISI	4.05 (2.97)	1.2–10.5	10.02 (5.97)	3.3–21.4	*F*_1,41_ = 22.97	<0.001***	0.36
Phi Total	36.36 (15.71)	18.3–93.2	28.69 (7.46)	16.0–41.2	*F*_1,41_ = 4.14	0.05*	0.09
**Striatal D2R BP**_**ND**_	10.12 (1.34)	8.2–13.4	10.20 (1.20)	8.6–12.3	*F*_1,38_ = 1.98	0.17	0.05
**DRD**_**AuC**_	.52 (.27)	.14–.98	.55 (.29)	.05–.98	*F*_1,39_ = 0.00	0.97	0.00
**PRD**_**AuC**_	.22 (.13)	.02–.50	.20 (.11)	.02–.42	*F*_1,39_ = 0.56	0.46	0.01
**Gender Distribution**	23 Female, 4 Male		15 Female, 5 Male		*χ* ^2^(1, *N* = 47) = 0.75	0.39	0.13
**Ethnic Distribution**	13 Wh, 13 Bl, 1 Hi		16 Wh, 2 Bl, 1 Hi, 1 Biracial		*χ* ^2^(1, *N* = 47) = 3.41	0.07^†^	0.27

BMI, body mass index; ISI, insulin sensitivity index; DRD, delayed reward discounting; PRD, probabilistic reward discounting; D2R, dopamine D2 receptor; BP_ND_, non-displaceable binding potential; NAc, nucleus accumbens; Wh, white; Bl, black; Hi, Hispanic.

*, *p*≤0.05

**, *p*≤0.01

***, *p*≤.001

^**†**^, *p* = 0.07 for comparison between obese and non-obese

### Relations of BMI and PBF to reward choice

BMI did not significantly relate to DRD_AuC_ within the total sample, non-obese, or obese individuals (**Table 2**). When PBF was entered as a predictor in place of BMI, it was not significantly related to DRD_AuC_ within the total sample or obese participants. The significance level for this relationship in non-obese individuals was *p* = 0.05 but did not survive Bonferroni multiple comparisons correction (**Table 2**).

**Table 2:** Hierarchical multiple linear regression analyses results in non-obese and obese men and women for delayed reward discounting (DRD_AuC_).

	*N*	Partial *r* for DRD_AuC_ and Predictor Variable	*F* for change in *R* ^*2*^, *p*-value	Effect Size (Cohen’s *f* ^*2*^)
**Body Mass Index**				
Total sample	45	-.05	.08, *p* = 0.78	.01
Non-obese	19	-.39	2.35, *p* = 0.15	.19
Obese	26	.01	.00, *p* = 0.98	.00
**Percent Body Fat**				
Total sample	45	-.05	2.70, *p* = 0.11	.08
Non-obese	19	-.52	4.69, ***p* = 0.05** ^#^	.36
Obese	26	-.01	.00, *p* = 0.97	.00
**Disposition Index**				
Total sample	45	.38	6.21, ***p* = 0.02** *	.17
Non-obese	19	.43	2.91, *p* = 0.11	.22
Obese	26	.43	4.48, ***p* = 0.05** ^#^	.22
**Striatal D2 Receptor Binding**				
Total sample	42	-.28	3.00, ***p* = 0.09** ^**†**^	.09
Non-obese	19	.05	.04, *p* = 0.85	.00
Obese	23	-.56	7.64, ***p* = 0.01** **	.45

*, *p*<0.05

**, *p* = 0.01

^**†**^, *p*<0.10

^**#**^, *p*≤0.05 but does not survive Bonferroni-corrected significance level (*α* = 0.025)

Neither BMI nor PBF were related to PRD_AuC_ in the total sample, non-obese, or obese groups (**Table 3**).

**Table 3:** Hierarchical multiple linear regression analyses results in non-obese and obese men and women for probabilistic reward discounting (PRD_AuC_).

	*N*	Partial *r* for PRD_AuC_ and Predictor Variable	*F* for change in *R* ^*2*^, *p*-value	Effect Size (Cohen’s *f* ^*2*^)
**Body Mass Index**				
Total sample	45	.01	.01, *p* = 0.93	.00
Non-obese	19	-.11	.16, *p* = 0.70	.01
Obese	26	-.01	.00, *p* = 0.95	.00
**Percent Body Fat**				
Total sample	45	-.07	.21, *p* = 0.65	.01
Non-obese	19	-.29	1.19, *p* = 0.30	.10
Obese	26	-.01	.17, *p* = 0.69	.00
**Disposition Index**				
Total sample	45	-.05	.08, *p* = 0.78	.01
Non-obese	19	.20	.51, *p* = 0.49	.05
Obese	26	-.30	2.01, *p* = 0.17	.09
**Striatal D2 Receptor Binding**				
Total sample	42	-.23	1.86, *p* = 0.18	.06
Non-obese	19	.14	.26, *p* = 0.62	.02
Obese	23	-.48	5.15, ***p* = 0.04** ^#^	.30

^**#**^, *p*≤0.05 but does not reach Bonferroni-corrected significance level (*α* = 0.025)

### Relation of β-cell function to reward choice

**Fig 2:**
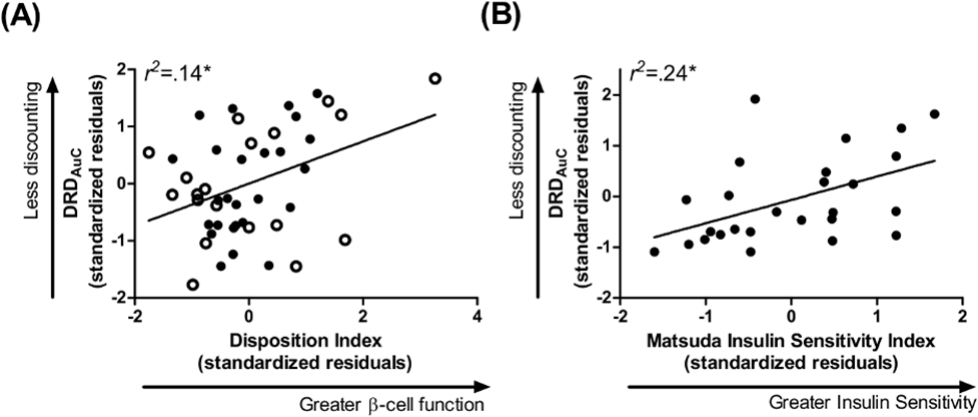
Insulin Function Relates to Delayed Reward Discounting in Total Sample and Obese Group. (A) β-cell function across the total sample and (B), insulin sensitivity in obese participants related to *greater* preference for a smaller but immediate monetary reward relative to one that was larger but delayed. Data points are standardized residuals of variables after controlling for age, gender, education, and ethnicity (and group in (A)). Clear data points, non-obese; filled data points, obese; DRD_AuC_, area under the curve for delayed reward discounting.

DI significantly related to DRD_AuC_ within the total sample (**Fig 2A**, **Table 2**and **S1 Table)**, such that individuals with higher β-cell function discounted delayed rewards at a lower rate than those with lower β-cell function. The significance level of this relationship in obese individuals was *p* = 0.05 but did not survive multiple comparison correction. In non-obese individuals, DI and DRD_AuC_ were not significantly related (**Table 2**and **S1 Table**).

In follow-up analyses, within the total sample, insulin sensitivity (Matsuda ISI) and DRD_AuC_ were related at a marginally significant level (**S2 Table**). This relationship was not significant in non-obese individuals but, in the obese group, lower insulin sensitivity was significantly associated with greater discounting and survived Bonferroni multiple comparisons correction (**Fig 2B, S2 Table**).

Insulin secretion (Phi Total) did not correlate with DRD_AuC_ in the total sample, obese, or non-obese groups (*F* for change in *R* ^*2*^≤2.39, *p*≥0.14).

DI was not significantly associated with PRD_AuC_ within the total sample or within non-obese or obese groups (**Table 3**)**.**

### Relation of striatal D2R BP_ND_ to reward choice

**Fig 3:**
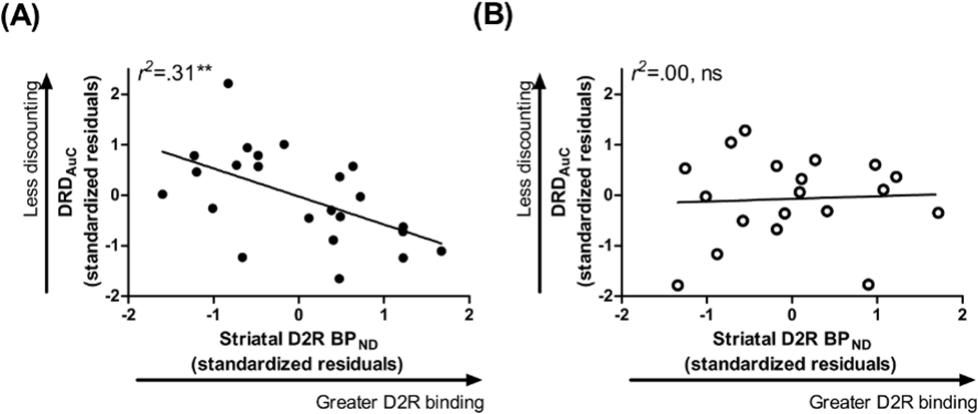
Striatal D2 Receptor Binding Relates to Delayed Reward Discounting in Obese but not Non-obese Individuals. (A) In obese individuals, higher striatal D2 receptor binding related to preference for a smaller, immediate monetary reward over a larger but delayed reward. (B) This relationship was not observed in non-obese individuals. Data points are standardized residuals of variables after controlling for age, gender, education, and ethnicity. DRD_AuC_, area under the curve for delayed reward discounting; D2R BP_ND_, dopamine D2 receptor specific binding.

Striatal D2R BP_ND_ significantly related to DRD_AuC_ in obese individuals (**Fig 3A**, **Table 2**, **S3 Table**), such that obese participants with higher striatal D2R BP_ND_ discounted delayed rewards to a higher degree than those with lower striatal D2R. This relationship was not significant in non-obese individuals (**Fig 3B**, **Table 2**and **S3 Table**). Within the total sample, the significance level of the relationship between striatal D2R BP_ND_ and DRD_AuC_ was trend-level (**Table 2**and **S3 Table**).

Follow-up analyses in the total sample indicated a non-significant trend correlating DRD_AuC_ and D2R BP_ND_ for putamen (*pr* = -0.31, *F* for change in *R* ^*2*^ = 3.59, *p* = 0.07) and non-significant for caudate and NAc (*F* for change in *R* ^*2*^≤2.15, *p*≥0.15). Within obese individuals, D2R binding in the putamen was significantly related to DRD_AuC_ (*pr* = -.57, *F* for change in *R* ^*2*^ = 8.18, *p* = 0≥.01, Cohen’s *f* ^*2*^ = .48) but not in the caudate or NAc (*F* for change in *R* ^*2*^≤4.44, *p*≥0.05). DRD_AuC_ did not relate to D2R BP_ND_ in any striatal region within non-obese individuals (*F* for change in *R* ^*2*^≤0.72, *p*≥0.41).

Striatal D2R did not relate to PRD_AuC_ in the total sample or in non-obese individuals (**Table 3)**. In obese individuals, the statistical significance of this relationship was *p* = 0.04 but did not survive multiple comparisons correction (**Table 3**).

### Relation of D2R BP_ND_ to BMI, PBF, and β-cell Function

Striatal D2R BP_ND_ did not relate to BMI, PBF, or DI in the total sample or within non-obese or obese groups (**Table 4**).

**Table 4:** Hierarchical multiple linear regression analyses results in non-obese and obese men and women for striatal D2 receptor (D2R) binding and other predictor variables.

	*N*	Partial *r* for D2R binding and Predictor Variable	*F* for change in *R* ^*2*^, *p*-value	Effect Size (Cohen’s *f* ^*2*^)
**Body Mass Index**				
Total sample	44	.05	.10, *p* = 0.76	.00
Non-obese	20	.10	.01, *p* = 0.72	.00
Obese	24	.00	.00, *p* = 0.99	.00
**Percent Body Fat**				
Total sample	44	.06	.15, *p* = 0.70	.00
Non-obese	20	.03	.01, *p* = 0.72	.00
Obese	24	.06	.06, *p* = 0.82	.00
**Disposition Index**				
Total sample	44	-.08	.26, *p* = 0.61	.00
Non-obese	20	-.18	.45, *p* = 0.51	.04
Obese	24	-.23	.96, *p* = 0.34	.05

### Within-subject Analyses in Women

**Fig 4:**
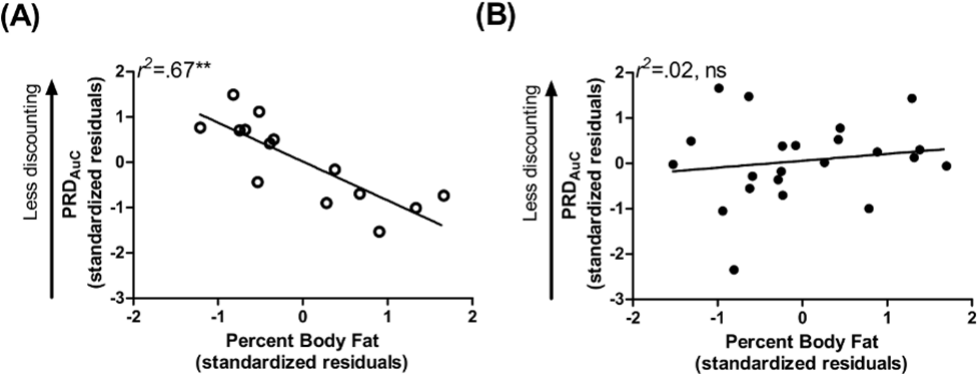
Body Fat Relates to Probabilistic Reward Discounting in Non-obese but not Obese Women. **(A)** In non-obese women, higher percent body fat related to *greater* preference for a smaller, certain monetary reward relative to one that was larger but less likely. This relationship was not observed in (B) obese women. Data points are standardized residuals of variables after controlling for age, education, and ethnicity. PRD_AuC_, area under the curve for probabilistic reward discounting.

In non-obese women, PBF significantly related to PRD_AuC_, such that non-obese women with higher PBF tended to prefer smaller but certain over larger but less likely monetary rewards (**S4 Table**, **Fig 4A**). This relationship was not observed in the total sample of women or within obese women (**S4 Table**, **Fig 4B**). Otherwise, relationships among variables were not particularly strengthened or weakened in analyses excluding men relative to the results described for analyses including both men and women (**S5–S7 Tables**).

## Discussion

The current study provides preliminary evidence of relationships between reward discounting behavior and 3 biological constructs related to obesity: PBF, pancreatic β-cell function, and striatal DA D2R binding in non-obese and obese humans. To our knowledge, this is the first human study of the relations between pancreatic insulin secretion and any type of discounting reward behavior in individuals carefully screened for prediabetes and diabetes. Further, in these same individuals, the relation between striatal D2R binding and reward discounting was characterized, unconfounded by diabetes or clinically significant addiction-like tendencies (i.e., binge eating disorder).

In line with our hypotheses, lower β-cell function across obese and non-obese individuals and lower insulin sensitivity in obese participants related to increased *delayed* reward discounting. One interpretation of these findings is that individuals with lower β-cell function and insulin sensitivity, which presumably reflect suboptimal insulin response to glucose overload, made more impulsive choices, choosing smaller but immediate receipt of a monetary reward over a delayed but larger reward amount. The mechanisms by which β-cell function and insulin sensitivity may relate to monetary reward discounting in humans remain to be discovered. Certainly, animal literature directly implicates insulin in regulation of brain reward circuitry [9–11] and behavior [11,14–17], and human neuroimaging studies show that insulin affects reward network activation and craving for food [3,18–25]. Overall, these previous reports indicate that insulin regulates appetitive behavior by decreasing craving and consumption of palatable food, possibly via its effects on DA transmission and network activity in reward-related brain regions. To our knowledge, the relationship of reward discounting to measures of β-cell function, insulin sensitivity, or insulin resistance has not been previously studied in animals or humans. Therefore, the relations between reward discounting and pancreatic β-cell function and insulin sensitivity observed here require replication.

The relationship we observed in non-obese women between greater *probabilistic* reward discounting and higher PBF is novel and indicates that women with higher PBF who are not obese may be more risk-averse since they preferred certain, smaller over less certain but larger monetary rewards. This finding as well as the near-significant relationship between PBF and *delayed* monetary reward discounting in non-obese women makes it tempting to speculate that non-obese women with relatively high body fat percentage, as observed here with monetary reward, may tend to consume foods that are easily accessible (more certain and more immediate) such as fast food relative to healthier meals that require time to plan and prepare. Alternatively, individuals who are more risk-averse may maintain healthy BMI despite preference for high-fat foods due to restrained (non-impulsive)_consumption of these foods. Interestingly, relationships between PBF and either type of monetary reward discounting were not observed in *obese* women. Future studies may determine whether transition to obesity in non-obese individuals with high PBF disrupts the relationship between this characteristic and reward discounting.

Contrary to our hypotheses, higher, rather than lower, striatal D2R binding was correlated with higher rates of delayed monetary reward discounting in obese individuals. That is, obese individuals with higher striatal D2R binding preferred smaller but immediate over larger but delayed monetary rewards. Our hypothesis was based on previous studies in animals and humans that demonstrate lower striatal D2-like receptor availability in obesity, addiction, and impulsivity [1, 27–29]. Further, administration of the stimulants d-amphetamine and methylphenidate decreases delay discounting in healthy individuals [61] and individuals with criminal and non-stimulant substance abuse histories [62], respectively. Some human studies have failed to find effects of pharmacological DA D2-like receptor agonism [63–64] or antagonism [65] on delayed discounting in healthy volunteers [64–65] and smokers [63]. However, in accordance with our finding, some previous studies do demonstrate that increased DA signaling and/or D2-like receptor availability relate to steeper delayed reward discounting. Administration of the DA precursor L-dopa increases delayed reward discounting in healthy individuals [65]. In addition, pharmacologic DA replacement or agonist therapy augments delayed reward discounting in Parkinson disease patients [66–67] and antagonism of D2R with metoclopramide decreases discounting in healthy individuals [68]. Also in line with our observation, addiction-like eating behavior is associated with increased DA signaling, as represented by individual multilocus genetic profile scores that account for variability in *ANKK1* alleles A2 and A1 [69], which are associated with higher and lower D2-like receptor binding, respectively [70]. Finally, we very recently found that, in many of the same participants studied here, higher rates of emotional eating relate to higher striatal D2R binding across both non-obese and obese groups, independent of BMI [71]. Some propose that striatal DA signaling may encode temporal information about reward receipt and therefore influence the subjective value of reward, such that relatively high DA signaling may imbue an immediate, smaller reward with greater subjective value than a delayed, larger one [65]. Assuming that higher striatal D2R binding reflects increased striatal DA transmission, our finding is in agreement with this hypothesis. Clearly, the relationship between striatal DA transmission, including D2-like receptor availability or D2R binding, and reward discounting may differ based on the population under study. It would be informative if future studies determined whether severity of disease (i.e. moderate obesity vs. morbid obesity or substance abuse vs. substance dependence) modulates the direction of the relationship between striatal DA transmission and reward discounting.

Interestingly, striatal D2R binding in *non-obese* individuals did not relate to delayed or probabilistic monetary reward discounting. Lower caudate BOLD response to palatable food has been associated with self-reported impulsivity in overweight but not healthy weight individuals [72]. The specificity of our finding and this fMRI study [72] to obese and overweight, respectively, as opposed to non-obese and normal-weight individuals, may indicate that DA signaling or striatal activation is more strongly coupled to impulsivity or discounting in individuals with a propensity for subclinical abnormal eating habits, such as increased desire for immediate gratification. The question of whether this coupling arises as a result of weight gain or predisposes individuals to overeating deservers further study, preferably in a longitudinal study that tracks aspects of DA signaling and reward discounting and/or impulsivity during weight gain or loss.

Importantly, when we broke down our analyses, D2R binding in dorsal striatal regions related more strongly to discounting behavior than D2R binding in ventral striatum (*p* = 0.10). We may not have had power to detect this relationship, since D2R binding is lower and PET measurements are therefore more variable in ventral relative to dorsal striatum. Although several human neuroimaging studies link discounting behavior, including subjective value of delayed monetary reward [73–74] and coding of reward magnitude [75] to ventral striatal reactivity, the dorsal striatum is thought to be involved in future reward prediction, with a particular role in encoding temporal delay to reward [76]. For example, greater caudate BOLD activation relates to increased discounting for reward receipt that is delayed one year relative to delays of less than one year [77]. Further, greater delay discounting correlates with dorsal locations relative to ventral locations of peak brain activation in caudate [78]. Intriguingly, dorsal and ventral striatal DA transmission or activation may differentially contribute to delay discounting and these relationships may depend on the population under study. For example, steeper discounting is associated with decreased ventral striatal DA release and D2-like receptor activation in pathological gamblers but correlated with greater dorsal striatal DA terminal function in individuals with Parkinson disease [79].

Notably, neither β-cell function nor striatal D2R binding were related to *probabilistic* reward discounting in non-obese or obese participants. The parameters of our probabilistic discounting task (i.e., large and small reward amounts, probability of receipt), may not have provided adequate sensitivity to detect individual differences in discounting tendencies. In both non-obese and obese groups, the range of PRD_AuC_ values was much less than that of DRD_AuC_ values (**Table 1**), and this lack of variability, coupled with small sample size, may have obscured any relationship with our measures of insulin function and D2R binding. Alternatively, the underlying processes for delayed and probabilistic discounting may not be identical [80]. For example, in probabilistic discounting, larger reward amounts are discounted equally or more than smaller amounts whereas larger amounts are discounted less in delay discounting [81–82].

Although higher PBF related to greater probabilistic reward discounting in non-obese women, non-obese and obese individuals did not differ significantly in delayed or probabilistic monetary reward discounting. Our results are similar to those of a previous study [52], in which obese women with binge eating disorder showed steeper discounting compared to obese without binge eating disorder and non-obese women, who did not differ in rates of delayed or probabilistic reward discounting, and the two types of discounting were positively correlated with each other. In other words, preference for immediate reward was related to preference for certain receipt of reward, as was true across our total sample, who did not have binge eating disorder. Our findings do contrast with those of three prior studies in which obese women demonstrated higher rates of delay discounting relative to normal-weight or lean women [53,83–84]. In one of these studies [83], statistical analyses did not account for significant age differences between the obese and normal weight groups, which is problematic because age affects reward discounting [56]. More importantly, the findings were *not* significant after controlling for education level; this is critical because the obese and normal weight groups differed significantly in education [83], and education relates to discounting rates [58]. In the other study [84], the delayed monetary reward was larger ($1,000) and the delay longer (up to 10 years). Therefore, differences in task parameter magnitudes may explain our differing results. These earlier studies also do not specify whether and how (i.e., self-report vs. blood test) individuals were screened for prediabetes or type 2 diabetes and none quantified β-cell function, insulin sensitivity, or insulin secretion. Perhaps differences in discounting between obese and non-obese groups may be more apparent in obese individuals with more severe metabolic or psychological pathology.

The lack of a relationship between BMI or PBF and striatal D2R binding is in agreement with findings from our previous study which did not detect differences in striatal binding between obese and non-obese individuals (some of whom are included in the present data analyses) [32]. Importantly, even when two individuals with BMI>25 in the non-obese group were excluded from the analyses, D2R binding was not different between this ‘true’ normal-weight group and the obese group. In contrast, our present results conflict with other previous studies. For example, striatal D2-like receptor binding was lower in obese relative to leaner individuals in some studies [1, 85–86]. However, others have found either higher striatal D2-like receptor binding in obese individuals [3, 87] or no relationship between BMI and striatal D2-like receptor binding [88–89]. These different findings may be due to several factors. First, the radioligands commonly used in these studies, including [^11^C]raclopride and [^18^F]fallypride, do not distinguish between D2 and D3 receptors [90] and may be displaced by endogenous DA [91–92]. The PET radioligand we used, [^11^C]NMB, overcomes these limitations: it is highly selective for D2 over D3 receptors [30] and is not displaceable by endogenous DA [31]. Thus, we measured D2R dopamine receptor binding whereas other studies measured availability of D2 and D3 confounded by status of endogenous dopamine, which can be influenced by environmental context. Differences in group characteristics across studies also may produce discordant results. Our non-obese and obese groups were rigorously screened for diabetes, psychiatric disorders, and other conditions that may affect DA transmission, thereby limiting confounding variables. Another explanation for disparate findings is that DA signaling may differ as a function of BMI severity. As others have speculated [88–89, 93], striatal DA system over-activity induced by over-eating in less severe forms of overweight or obesity may eventually downregulate striatal D2-like receptor binding, as observed in extremely obese individuals [1, 86]. Alternatively, obese individuals with higher striatal D2-like receptor binding at baseline may be less prone to developing more severe obesity or eating pathology. The longitudinal study of clinical eating or metabolic abnormalities, including binge eating disorder and diabetes may help elucidate if, how, and when metabolic health and DA signaling function interact to affect reward behavior. These possible relationships certainly merit further study, given their potential roles in reward-related regulation of food consumption.

Finally, the results of the present study suggest that striatal D2R binding does not relate directly to β-cell function or BMI. The former finding is unexpected given strong evidence that insulin and DA interact to affect appetitive behavior in nonhuman animals [11–13]. Therefore, we believe our null finding is likely due to the particular measures (D2R binding, DI) and experimental conditions (i.e. single time point) employed in our research design. Other features of striatal DA signaling, including synaptic neurotransmission and transport, may be related to measures of insulin secretion, sensitivity, or resistance. Other measures of insulin function and other metabolic variables (i.e. ghrelin, leptin) may mediate a relationship between D2R binding and DI and/or reward discounting behavior. In addition, other central neurotransmitter systems, including serotonergic and noradrenergic, and other brain regions, including hypothalamic, prefrontal cortical and subcortical limbic regions, may interact with insulin function and DA signaling to affect reward discounting behavior (for review, see [47]).

Despite the methodological strengths of the current study, the results are correlational in nature. For example, low β-cell function may influence behavior or, conversely, individuals with altered reward behavior may have poor eating habits that lead to poor β-cell function. Longitudinal or interventional studies of changes in β-cell function or weight will be important for understanding the direction of these relations. In addition, while we did include gender as a covariate in regression analyses, we did not directly measure hormone levels in women to determine menstrual phase, which may interact with cortical DA activity to affect DA-dependent working memory [94] and delay discounting [95]. A number of women in our sample reported absent or irregular menstrual cycle due to contraceptive method and hysterectomy (*n =* 10). Therefore, due to variability in contraceptive type, and lack of hormone levels to determine menstrual phase during the PET scans, we cannot assess whether menstrual phase contributed to our results, including differences between non-obese and obese individuals. Finally, replication and validation of our findings are necessary due to the novel nature of our findings and our ultimately small sample size, which likely increased Type II error.

Overall, our results provide initial support for the hypotheses that metabolic health, pancreatic β-cell function and striatal D2R binding relate to monetary reward discounting behavior in humans. Specifically, higher PBF in non-obese women was associated with risk-aversion as indicated by greater tendency to prefer certain, smaller over larger but less likely monetary reward. Worse β-cell function in the total sample and lower insulin sensitivity in obese participants correlated with preference for smaller, immediate over delayed, larger monetary rewards, an indication of greater impulsivity. Higher striatal D2R binding also related to greater delay discounting in obese individuals. We found no significant relations between β-cell function and striatal D2R binding in obese or non-obese individuals. Whether and how insulin and DA signaling interact to affect reward discounting behavior in humans clearly deserves further study, as many different aspects of both of these systems other than those studied here (β-cell function and D2R binding) exist. Replication and extension of our findings by investigation of other aspects of DA signaling and insulin function will lend support to the notion that these variables interact in humans to regulate reward-related aspects of eating behavior.

## Supporting Information

S1 Table Summary of hierarchical multiple linear regression analyses for prediction of delayed monetary reward discounting (DRD_AuC_) by disposition index (DI) in the total sample, non-obese, and obese individuals. (DOCX)

S2 Table Summary of hierarchical multiple linear regression analyses for prediction of delayed monetary reward discounting (DRD_AuC_) by Matsuda insulin sensitivity indices (ISI) in the total sample, non-obese, and obese individuals. (DOCX)

S3 Table Summary of hierarchical multiple linear regression analyses for prediction of delayed monetary reward discounting (DRD_AuC_) by striatal D2 receptor (D2R) binding in the total sample, non-obese, and obese individuals. (DOCX)

S4 Table Summary of hierarchical multiple linear regression analyses for prediction of probabilistic monetary reward discounting (PRD_AuC_) by percent body fat (PBF) in women. (DOCX)

S5 Table Hierarchical multiple linear regression analyses results in non-obese and obese women for delayed reward discounting (DRD_AuC_). (DOCX)

S6 Table Hierarchical multiple linear regression analyses results in non-obese and obese women for probabilistic reward discounting (PRD_AuC_). (DOCX)

S7 Table Hierarchical multiple linear regression analyses results in non-obese and obese women for striatal D2 receptor (D2R) binding and other predictor variables. (DOCX)
