# Correction: Insulin, Central Dopamine D2 Receptors, and Monetary Reward Discounting in Obesity

**DOI:** 10.1371/journal.pone.0147063

**Published:** 2016-01-08

**Authors:** Sarah A. Eisenstein, Danuta M. Gredysa, Jo Ann Antenor–Dorsey, Leonard Green, Ana Maria Arbeláez, Jonathan M. Koller, Kevin J. Black, Joel S. Perlmutter, Stephen M. Moerlein, Tamara Hershey

The following information is missing from the Funding section: This study was also supported by grant NIH P30 DK056341 from the Washington University Nutrition Obesity Research Center.

## References

[1] Eisenstein SA, Gredysa DM, Antenor–Dorsey JA, Green L, Arbeláez AM, Koller JM, et al (2015) Insulin, Central Dopamine D2 Receptors, and Monetary Reward Discounting in Obesity. PLoS ONE 10(7): e0133621 doi: 10.1371/journal.pone.0133621 26192187 10.1371/journal.pone.0133621 PMC4507849

