# Supplementary material for: Insulin, Central Dopamine D2 Receptors, and Monetary Reward Discounting in Obesity
Source: PLoS One. 2015 Jul 20;10(7):e0133621. doi: 10.1371/journal.pone.0133621 (PMC4507849; doi:10.1371/journal.pone.0133621)
Supplement: S1 Table — (DOCX) [file pone.0133621.s001.docx]

| **Table S1.** Summary of hierarchical multiple linear regression analyses for prediction of delayed monetary reward discounting (DRD_AuC_) by disposition index (DI) in the total sample, non-obese, and obese individuals. | | | | | | | |
| --- | --- | --- | --- | --- | --- | --- | --- |
| **DI and DRD_AuC_** | Step 1 | | |  | Step 2 | | |
| **Total sample (*N* =45)** |  |  |  |  |  |  |  |
| Variable | *B* | *SE B* | *β* |  | *B* | *SE B* | *β* |
| Age | -.01 | .01 | -.11 |  | -.01 | .01 | -.25 |
| Gender | -.02 | .11 | -.02 |  | .05 | .11 | .07 |
| Education | .03 | .03 | .18 |  | .02 | .03 | .14 |
| White or not | .01 | .20 | .01 |  | .07 | .19 | .07 |
| Group | .00 | .09 | .01 |  | .17 | .11 | .32 |
|  |  |  |  |  |  |  |  |
| Disposition Index |  |  |  |  | .00 | .00 | **.48*** |
|  |  |  |  |  |  |  |  |
| *R^2^* |  | .05 |  |  |  | .19 |  |
| *F* for change in *R^2^* |  | .44, *p*=0.82 |  |  | 6.21, *p*=0.02 (Cohen’s *f^2^*=0.17) | | |
| **Non-obese (*n*=19)** |  |  |  |  |  |  |  |
| Variable | *B* | *SE B* | *β* |  | *B* | *SE B* | *β* |
| Age | .00 | .02 | .07 |  | -.01 | .02 | -.23 |
| Gender | .06 | .17 | .10 |  | .16 | .17 | .25 |
| Education | .01 | .07 | .02 |  | -.01 | .06 | -.03 |
| White or not | .30 | .44 | .22 |  | .45 | .42 | .33 |
|  |  |  |  |  |  |  |  |
| Disposition Index |  |  |  |  | .00 | .00 | .54 |
|  |  |  |  |  |  |  |  |
| *R^2^* |  | .06 |  |  |  | .23 |  |
| *F* for change in *R^2^* |  | .23, *p*=0.92 |  |  | 2.91, *p*=0.11 (Cohen’s *f^2^*=0.22) | | |
| **Obese (*n*=26)** |  |  |  |  |  |  |  |
| Variable | *B* | *SE B* | *β* |  | *B* | *SE B* | *β* |
| Age | -.01 | .01 | -.21 |  | -.01 | .01 | -.28 |
| Gender | -.09 | .16 | -.12 |  | -.02 | .15 | -.03 |
| Education | .03 | .03 | .19 |  | .01 | .03 | .08 |
| White or not | -.04 | .24 | -.04 |  | -.02 | .22 | -.02 |
|  |  |  |  |  |  |  |  |
| Disposition Index |  |  |  |  | .00 | .02 | **.44^#^** |
|  |  |  |  |  |  |  |  |
| *R^2^* |  | .12 |  |  |  | .28 |  |
| *F* for change in *R^2^* |  | .70, *p*=0.60 |  |  | 4.48, *p*=0.05 (Cohen’s *f^2^*=0.22) | | |
| *****, *p*=0.02; **^#^**, *p*≤0.05 but does not survive Bonferroni-corrected significance level (*α*=0.025) | | | | | | | |
