# Supplementary material for: Insulin, Central Dopamine D2 Receptors, and Monetary Reward Discounting in Obesity
Source: PLoS One. 2015 Jul 20;10(7):e0133621. doi: 10.1371/journal.pone.0133621 (PMC4507849; doi:10.1371/journal.pone.0133621)
Supplement: S2 Table — (DOCX) [file pone.0133621.s002.docx]

| **Table S2.** Summary of hierarchical multiple linear regression analyses for prediction of delayed monetary reward discounting (DRD_AuC_) by Matsuda insulin sensitivity indices (ISI) in the total sample, non-obese, and obese individuals. | | | | | | | |
| --- | --- | --- | --- | --- | --- | --- | --- |
| **ISI and DRD_AuC_** | Step 1 | | |  | Step 2 | | |
| **Total sample (*N* =45)** |  |  |  |  |  |  |  |
| Variable | *B* | *SE B* | *β* |  | *B* | *SE B* | *β* |
| Age | -.01 | .01 | -.11 |  | -.01 | .01 | -.19 |
| Gender | -.02 | .11 | -.02 |  | .02 | .11 | .03 |
| Education | .03 | .03 | .18 |  | .02 | .03 | .13 |
| White or not | .01 | .20 | .01 |  | .07 | .20 | .06 |
| Group | .00 | .09 | .01 |  | .14 | .11 | .25 |
|  |  |  |  |  |  |  |  |
| Matsuda ISI |  |  |  |  | .02 | .01 | **.37^†^** |
|  |  |  |  |  |  |  |  |
| *R^2^* |  | .05 |  |  |  | .14 |  |
| *F* for change in *R^2^* |  | .44, *p*=0.82 |  |  | 3.57, *p*=0.07 (Cohen’s *f^2^*=0.10) | | |
| **Non-obese (*n*=19)** |  |  |  |  |  |  |  |
| Variable | *B* | *SE B* | *β* |  | *B* | *SE B* | *β* |
| Age | .00 | .02 | .07 |  | .00 | .02 | -.03 |
| Gender | .06 | .17 | .10 |  | .09 | .17 | .14 |
| Education | .01 | .07 | .02 |  | -.01 | .07 | -.03 |
| White or not | .30 | .44 | .22 |  | .35 | .45 | .26 |
|  |  |  |  |  |  |  |  |
| Matsuda ISI |  |  |  |  | .01 | .01 | .22 |
|  |  |  |  |  |  |  |  |
| *R^2^* |  | .06 |  |  |  | .10 |  |
| *F* for change in *R^2^* |  | .23, *p*=0.92 |  |  | .51, *p*=0.49 (Cohen’s *f^2^*=0.04) | | |
| **Obese (*n*=26)** |  |  |  |  |  |  |  |
| Variable | *B* | *SE B* | *β* |  | *B* | *SE B* | *β* |
| Age | -.01 | .01 | -.21 |  | -.01 | .01 | -.25 |
| Gender | -.09 | .16 | -.12 |  | -.01 | .15 | -.02 |
| Education | .03 | .03 | .19 |  | .01 | .03 | .08 |
| White or not | -.04 | .24 | -.04 |  | .05 | .22 | .04 |
|  |  |  |  |  |  |  |  |
| Matsuda ISI |  |  |  |  | .05 | .02 | **.50*** |
|  |  |  |  |  |  |  |  |
| *R^2^* |  | .12 |  |  |  | .33 |  |
| *F* for change in *R^2^* |  | .70, *p*=0.60 |  |  | 6.35, *p*=0.02 (Cohen’s *f^2^*=0.31) | | |
| *, *p*<0.05; **^†^**, *p*<0.10 | | | | | | | |
