# Supplementary material for: Insulin, Central Dopamine D2 Receptors, and Monetary Reward Discounting in Obesity
Source: PLoS One. 2015 Jul 20;10(7):e0133621. doi: 10.1371/journal.pone.0133621 (PMC4507849; doi:10.1371/journal.pone.0133621)
Supplement: S3 Table — (DOCX) [file pone.0133621.s003.docx]

| **Table S3.** Summary of hierarchical multiple linear regression analyses for prediction of delayed monetary reward discounting (DRD_AuC_) by striatal D2 receptor (D2R) binding in the total sample, non-obese, and obese individuals. | | | | | | | |
| --- | --- | --- | --- | --- | --- | --- | --- |
| **Striatal D2R and DRD_AuC_** | Step 1 | | |  | Step 2 | | |
| **Total sample (*N* =42)** |  |  |  |  |  |  |  |
| Variable | *B* | *SE B* | *β* |  | *B* | *SE B* | *β* |
| Age | -.00 | .01 | -.07 |  | -.01 | .01 | -.20 |
| Gender | -.01 | .11 | -.02 |  | -.07 | .11 | -.11 |
| Education | .02 | .03 | .14 |  | .04 | .03 | .24 |
| White or not | -.05 | .21 | -.05 |  | .06 | .21 | .06 |
| Group | -.02 | .10 | -.03 |  | .02 | .10 | .04 |
|  |  |  |  |  |  |  |  |
| Striatal D2R |  |  |  |  | -.08 | .05 | **-.36^†^** |
|  |  |  |  |  |  |  |  |
| *R^2^* |  | .03 |  |  |  | .11 |  |
| *F* for change in *R^2^* |  | .24, *p*=0.95 |  |  | 3.00, *p*=0.09 (Cohen’s *f*^2^=.09) | | |
| **Non-obese (*n*=19)** |  |  |  |  |  |  |  |
| Variable | *B* | *SE B* | *β* |  | *B* | *SE B* | *β* |
| Age | .00 | .02 | .07 |  | .00 | .02 | .08 |
| Gender | .06 | .17 | .10 |  | .09 | .21 | .14 |
| Education | .01 | .07 | .02 |  | .00 | .07 | .01 |
| White or not | .30 | .44 | .22 |  | .25 | .51 | .18 |
|  |  |  |  |  |  |  |  |
| Striatal D2R |  |  |  |  | .02 | .11 | .08 |
|  |  |  |  |  |  |  |  |
| *R^2^* |  | .06 |  |  |  | .06 |  |
| *F* for change in *R^2^* |  | .23, *p*=0.92 |  |  | .04, *p*=0.85 (Cohen’s *f*^2^=.00) | | |
| **Obese (*n*=23)** |  |  |  |  |  |  |  |
| Variable | *B* | *SE B* | *β* |  | *B* | *SE B* | *β* |
| Age | -.01 | .01 | -.17 |  | -.02 | .01 | -.44 |
| Gender | -.09 | .16 | -.13 |  | -.11 | .14 | -.16 |
| Education | .02 | .03 | .12 |  | .05 | .03 | ..36 |
| White or not | -.15 | .25 | -.15 |  | -.03 | .22 | -.03 |
|  |  |  |  |  |  |  |  |
| Striatal D2R |  |  |  |  | -.13 | .05 | **-.68**** |
|  |  |  |  |  |  |  |  |
| *R^2^* |  | .10 |  |  |  | .38 |  |
| *F* for change in *R^2^* |  | .49, *p*=0.75 |  |  | 7.64, *p*=0.01 (Cohen’s *f*^2^=.45) | | |
| **, *p*<0.01 |  |  |  |  |  | | |
