# Supplementary material for: Insulin, Central Dopamine D2 Receptors, and Monetary Reward Discounting in Obesity
Source: PLoS One. 2015 Jul 20;10(7):e0133621. doi: 10.1371/journal.pone.0133621 (PMC4507849; doi:10.1371/journal.pone.0133621)
Supplement: S4 Table — (DOCX) [file pone.0133621.s004.docx]

| **Table S4.** Summary of hierarchical multiple linear regression analyses for prediction of probabilistic monetary reward discounting (PRD_AuC_) by percent body fat (PBF) in women. | | | | | | | |
| --- | --- | --- | --- | --- | --- | --- | --- |
| **PBF and PRD_AuC_** | Step 1 | | |  | Step 2 | | |
| **Total sample (*N* =36)** |  |  |  |  |  |  |  |
| Variable | *B* | *SE B* | *β* |  | *B* | *SE B* | *β* |
| Age | .00 | .00 | .05 |  | .00 | .00 | .06 |
| Education | .02 | .01 | .32 |  | .02 | .01 | .31 |
| White or not | -.11 | .10 | -.22 |  | -.10 | .10 | -.20 |
| Group | .03 | .05 | .12 |  | .08 | .09 | .30 |
|  |  |  |  |  |  |  |  |
| Percent Body Fat |  |  |  |  | .00 | .01 | -.20 |
|  |  |  |  |  |  |  |  |
| *R^2^* |  | .10 |  |  |  | .11 |  |
| *F* for change in *R^2^* |  | .85, *p*=0.51 |  |  | 0.35, *p*=0.56 (Cohen’s *f^2^*=0.01) | | |
| **Non-obese (*n*=14)** |  |  |  |  |  |  |  |
| Variable | *B* | *SE B* | *β* |  | *B* | *SE B* | *β* |
| Age | .00 | .01 | .04 |  | .01 | .01 | .23 |
| Education | -.02 | .03 | -.27 |  | -.05 | .02 | -.67 |
| White or not | .36 | .23 | .62 |  | .70 | .16 | 1.19 |
|  |  |  |  |  |  |  |  |
| Percent Body Fat |  |  |  |  | -.02 | .00 | **-.84**** |
|  |  |  |  |  |  |  |  |
| *R^2^* |  | .22 |  |  |  | .75 |  |
| *F* for change in *R^2^* |  | .92, *p*=0.47 |  |  | 18.67, *p*=0.002 (Cohen’s *f^2^*=2.12) | | |
| **Obese (*n*=22)** |  |  |  |  |  |  |  |
| Variable | *B* | *SE B* | *β* |  | *B* | *SE B* | *β* |
| Age | .00 | .00 | .17 |  | .00 | .00 | .15 |
| Education | .03 | .02 | .47 |  | .03 | .02 | .47 |
| White or not | -.24 | .11 | -.49 |  | -.24 | .11 | -.48 |
|  |  |  |  |  |  |  |  |
| Percent Body Fat |  |  |  |  | .01 | .01 | .13 |
|  |  |  |  |  |  |  |  |
| *R^2^* |  | .27 |  |  |  | .29 |  |
| *F* for change in *R^2^* |  | 2.25, *p*=0.12 |  |  | .43, *p*=0.52 (Cohen’s *f^2^*=0.03) | | |
| **, *p*<0.01 | | | | | | | |
