# Supplementary material for: Insulin, Central Dopamine D2 Receptors, and Monetary Reward Discounting in Obesity
Source: PLoS One. 2015 Jul 20;10(7):e0133621. doi: 10.1371/journal.pone.0133621 (PMC4507849; doi:10.1371/journal.pone.0133621)
Supplement: S5 Table — (DOCX) [file pone.0133621.s005.docx]

| **Table S5.** Hierarchical multiple linear regression analyses results in non-obese and obese women for delayed reward discounting (DRD_AuC_). | | | | |
| --- | --- | --- | --- | --- |
|  | *N* | Partial *r* for DRD_AuC_ and Predictor Variable | *F* for change in *R^2^*, *p*-value | Effect Size (Cohen’s *f^2^*) |
| **Body Mass**  **Index** | | | | |
| Total sample | 36 | .04 | .05, *p*=0.82 | .00 |
| Non-obese | 14 | -.51 | 3.13, *p*=0.11 | .35 |
| Obese | 22 | .10 | .16, *p*=0.70 | .01 |
| **Percent Body**  **Fat** | | | | |
| Total sample | 36 | -.17 | 0.93, *p*=0.34 | .03 |
| Non-obese | 14 | -.64 | 6.25, ***p*=0.03^#^** | .70 |
| Obese | 22 | .11 | .20, *p*=0.66 | .01 |
| **Disposition**  **Index** | | | | |
| Total sample | 36 | .38 | 5.11, ***p*=0.03*** | .17 |
| Non-obese | 14 | .38 | 1.49, *p*=0.25 | .17 |
| Obese | 22 | .43 | 3.78, ***p*=0.07^†^** | .22 |
| **Striatal D2**  **Receptor**  **Binding** | | | | |
| Total sample | 33 | -.28 | 2.27, *p*=0.14 | .08 |
| Non-obese | 14 | .06 | .03, *p*=0.86 | .00 |
| Obese | 19 | -.53 | 5.59, ***p*=0.03^#^** | .45 |
| *****, *p*<0.05; **^†^**, *p*<0.10; **^#^**, *p*≤0.05 but does not survive Bonferroni-corrected significance level (*α*=0.025) | | | | |
