# Supplementary material for: Insulin, Central Dopamine D2 Receptors, and Monetary Reward Discounting in Obesity
Source: PLoS One. 2015 Jul 20;10(7):e0133621. doi: 10.1371/journal.pone.0133621 (PMC4507849; doi:10.1371/journal.pone.0133621)
Supplement: S6 Table — (DOCX) [file pone.0133621.s006.docx]

| **Table S6.** Hierarchical multiple linear regression analyses results in non-obese and obese women for probabilistic reward discounting (PRD_AuC_). | | | | |
| --- | --- | --- | --- | --- |
|  | *N* | Partial *r* for PRD_AuC_ and Predictor Variable | *F* for change in *R^2^*, *p*-value | Effect Size (Cohen’s *f^2^*) |
| **Body Mass**  **Index** |  |  | | |
| Total sample | 36 | .05 | .07, *p*=0.79 | .00 |
| Non-obese | 14 | -.64 | 6.26, ***p*=0.03^#^** | .70 |
| Obese | 22 | .04 | .03, *p*=0.88 | .00 |
| **Percent Body**  **Fat** |  |  | | |
| Total sample | 36 | -.11 | .35, *p*=0.56 | .01 |
| Non-obese | 14 | -.82 | 18.67, ***p*<0.01**** | 2.07 |
| Obese | 22 | .16 | .43, *p*=0.52 | .03 |
| **Disposition**  **Index** |  |  | | |
| Total sample | 36 | -.04 | .04, *p*=0.84 | .00 |
| Non-obese | 14 | .63 | 5.81, ***p*=0.04^#^** | .65 |
| Obese | 22 | -.35 | 2.41, *p*=0.14 | .14 |
| **Striatal D2**  **Receptor Binding** |  |  | | |
| Total sample | 33 | -.21 | 1.29, *p*=0.27 | .05 |
| Non-obese | 14 | .09 | .07, *p*=0.81 | .01 |
| Obese | 19 | -.54 | 5.82, ***p*=0.03^#^** | .42 |
| ******, *p*<0.01;  **^#^**, *p*≤0.05 but does not reach Bonferroni-corrected significance level (*α*=0.025) | | | | |
