# Supplementary material for: Insulin, Central Dopamine D2 Receptors, and Monetary Reward Discounting in Obesity
Source: PLoS One. 2015 Jul 20;10(7):e0133621. doi: 10.1371/journal.pone.0133621 (PMC4507849; doi:10.1371/journal.pone.0133621)
Supplement: S7 Table — (DOCX) [file pone.0133621.s007.docx]

| **Table S7.** Hierarchical multiple linear regression analyses results in non-obese and obese women for striatal D2 receptor (D2R) binding and other predictor variables. | | | | |
| --- | --- | --- | --- | --- |
|  | *N* | Partial *r* for D2R binding and Predictor Variable | *F* for change in *R^2^*, *p*-value | Effect Size (Cohen’s *f^2^*) |
| **Body Mass**  **Index** |  |  | | |
| Total sample | 35 | .01 | .00, *p*=0.96 | .00 |
| Non-obese | 15 | .34 | 1.29, *p*=0.28 | .13 |
| Obese | 20 | -.08 | .10, *p*=0.76 | .01 |
| **Percent Body**  **Fat** |  |  | | |
| Total sample | 35 | .02 | .01, *p*=0.93 | .00 |
| Non-obese | 15 | -.02 | .01, *p*=0.94 | .00 |
| Obese | 20 | -.04 | .02, *p*=0.89 | .00 |
| **Disposition**  **Index** |  |  | | |
| Total sample | 35 | -.11 | .35, *p*=0.56 | .01 |
| Non-obese | 15 | .02 | .00, *p*=0.95 | .00 |
| Obese | 20 | -.23 | .85, *p*=0.37 | .06 |
